# The role of epithelial-mesenchymal transition in the post-lung transplantation bronchiolitis obliterans

**DOI:** 10.1186/s13019-017-0673-6

**Published:** 2017-12-20

**Authors:** Chong Zhang, Yuequn Niu, Li Yu, Wang Lv, Haichao Xu, Abudumailamu Abuduwufuer, Jinlin Cao, Jian Hu

**Affiliations:** Department of Thoracic Surgery, First Affiliated Hospital of Zhejiang University, No. 79 Qingchun Road, Zhejiang, Hangzhou 310003 China

**Keywords:** Lung transplantation, Chronic rejection, Bronchiolitis obliterans, Epithelial-mesenchymal transition, E-cadherin, Vimentin

## Abstract

**Background:**

Many patients who receive lung transplantation (LT) operations develop varying degrees of bronchiolitis obliterans (BO) after the surgeries. Epithelial-mesenchymal transition (EMT) is considered to be related to the process of bronchiolitis obliterans. In this study we simulated the pathological process of post-lung transplantation bronchiolitis obliterans, and explored the correlation between BO and EMT of small airway epithelial cells.

**Methods:**

We transplanted the left lungs of F344 rats to Lewis rats by the Tri-cuff anastomosis and established the allogeneic rat left lung orthotopic transplantation model. Cyclosporine and lipopolysaccharide were administrated appropriately after the surgery. The histological structure and the expression levels of the EMT markers was observed with the methods of HE staining, Masson staining and immunohistochemistry. The analysis of enumeration data was performed using Fisher’s Exact test and Spearman’s rank correlation was used for the correlation analysis.

**Results:**

Inflammatory cell infiltration, fibroplasia of bronchiole walls and significant lumen stenosis were found in the pulmonary mesenchyme of the transplanted lungs. The positive expression rate of E-cadherin in the transplanted lungs was 38.50% (5/13), significantly lower than that in the normal lung tissues [87.50% (7/8)] (*P* < 0.05), while the positive expression rate of Vimentin was 76.92% (10/13) which is significantly higher than that in the normal lung tissues [25.00% (2/8)] (*P* < 0.05). And a negative correlation existed between the expression levels of E-cadherin and Vimentin (*r* = −0.750, *P* < 0.01).

**Conclusions:**

In the disease model we established in this study, we found pathological changes that met BO characteristics happened in the transplanted lungs. Meanwhile, the small airway epithelial cells of transplanted lungs underwent an epithelial-mesenchymal transition, which indicated a role of EMT in the BO airway remodeling.

## Background

Up to now, lung transplantation (LT) is the only effective method in the treatment of terminal pulmonary disease, but chronic dysfunction of transplanted lungs related to chronic graft rejection limits the long-term effect of LT [1]. The development of chronic lung allograft dysfunction (CLAD), with the Bronchiolitis obliterans (BO) being the most common manifestation, is considered to be a major player in the process of chronic graft failure and the most frequent cause of long-term morbidity and mortality after LT [2, 3]. As a process of chronic airway rejection, BO is manifested in the fibroplasia and occlusion of small airway, leading to persistent decline of lung function. Approximately 68% of lung transplant patients showed varying degrees of BO within 3 months after surgery [4]. The structural alteration of the terminal bronchioles is a main cause of the airway obstruction in the process of BO [5]. Since the principal pathological change of BO is airway remodeling, inhibition of which could be the key of future clinical management of post-lung transplantation BO.

EMT refers to a process that epithelial cells lose their original epithelial features and obtain some mesenchymal characteristics, such as losing cell adhesion, gaining the ability to migrate and extending to a spindle shape similar to fibroblasts in morphology. Then the epithelial cells stretch out pseudopodia, separate themselves from the surrounding cells, break through the basement membrane and become new mesenchymal cells [6, 7]. In terms of surface markers, EMT is characterized by lost expression of E-cadherin, decreased expression of epithelial markers, and increased expression of mesenchymal markers such as Vimentin and smooth muscle actin (SMA) in cells [8]. E-cadherin, a calcium-dependent cell surface protein, which can promote the adhesion between the epithelial cells, is the main protein to anchor the adhesion junction between epithelial cells. The expression of E-cadherin decreases in the process of embryonic development, tissue fibrosis and cancer [9]. On the contrary, Vimentin, which is only found in mesenchymal cells, is a kind of intermediate filament protein, attached to nucleus, endoplasmic reticulum, and the sides or end of mitochondrion, making great contribution to support and anchor organelles in the protoplasm and playing an important role in the cell shape maintenance. [10]. Current studies have showed that EMT is involved in the BO airway remodeling [11, 12]. Thus, further exploration of the mechanism and pathological significance of EMT may provide novel targets for treatment of post-lung transplantation bronchiolitis obliterans.

In the present study, a left lung orthotopic transplantation model was successfully established in rats. We then further explored the histological structure changes in BO and detected the expression levels of the several essential EMT markers. We found EMT took part in the process of airway remodeling in the post-lung transplantation BO, which provided a potential target for the future treatment of BO after the lung transplantation surgery.

## Methods

Both male and female healthy SPF (specific pathogen free) F344 and Lewis rats, weighted 200-250 g, were included as the donors and recipients of the lung transplantation surgery. The weight of the donor and the corresponding recipient was controlled as close as possible. The rats were all fed in SPF environment. Temperature of the animal room was 20–22 °C and photoperiod was 12 h. The rats adapted themselves to the environment for a week before the experiments, drinking and eating freely. The ones with normal behavior and showed no adverse reactions were included in the surgery. The rats were divided into 2 groups. F344 rats with sham operations were grouped as normal control and Lewis rats received allogeneic left lung orthotopic transplantation [F344-to-Lewis] were grouped as the experimental group.

We established the allogeneic lung transplantation model. F344 rats (donors) with intraperitoneal injection of atropine 0.25 mg were anesthetized with intraperitoneal injection of chloral hydrate (10 vol%) 4 ml/kg, and connected to a small animal ventilator after the tracheal intubation. Then abdominal median incisions were made and the rats were injected with heparin 1000 U/kg via inferior vena cava. Next we cut open the diaphragm and the anterior chest wall to sufficiently expose the cardiopulmonary tissues. The lung lavage was performed using 4 °C improved Euro-Collins solution and the perfusion continued until the lung tissues were pale and the perfusates were clear and translucent. Then we separated the left lung hilum, sheared off the left pulmonary artery, left pulmonary vein and left bronchus, and fixed a cannula to each broken end by ligation. The donor lungs were preserved in 4 °C improved Euro-Collins solution and set aside.

Lewis rats (recipients) received the same operations of anesthesia, intubation and ventilation surgery as the donor rats. We got into the chest via an incision in the left fourth intercostal space and separated the hilum. Then we clamped the pulmonary artery and pulmonary vein by microvascular clamp and sheared them off from the distal end. Next, the pulmonary artery and pulmonary vein of the donor lung were inserted into the corresponding vessels of the recipient and fixed by ligation. Afterwards the vein and artery were opened in sequence. Then we clamped the bronchus, sheared it off from the distal end, removed the original lung and put the donor lung into the original position in the thoracic cavity. After that we inserted the bronchus of the donor lung into the corresponding broken end and fixed it by ligation. Finally the lung was ventilated to recover. The criterions of successful anastomosis: (1) no blood or air leaking from the three anastomotic stomas; (2) the pulmonary artery and pulmonary vein were both filled; (3) the lung presented as pink, uniform and elastic. Finally we put into the thoracic drainage tube and performed sternal closure and anesthesia recovery.

We randomly chose healthy F344 rats to perform sham operation, which only included the thoracotomy and sternal closure surgery while the lung transplantation was not performed. The operation of anesthesia, intubation, ventilation, incision cutting, sternal closure and anesthesia recovery were all the same as that of the recipients described previously.

Next, we established the BO model [13]. From the first day after the surgery, the rats from experimental group were given intraperitoneal injection of cyclosporine (5 mg/kg•d) for 10 days in order to reduce the acute rejection. At the 28th day after the surgery, the rats from experimental group were given intratracheal administration with lipopolysaccharide (0.5 mg/kg) while the treatment of the control group was intratracheal administration of PBS at the 28th day after the sham operation.

The rats from both control and experimental group were killed at the 90th day after the surgery and their left lungs were taken out and fixed by 10% Formalin. The paraffin sections of the samples were made and examined by HE staining, Masson staining and immunohistochemistry.

Following dewaxing and hydration according to the normal protocols, HE staining and Masson staining were performed. After mounting, the histological structure was observed and the differences between experimental group and control group were compared.

Streptavidin-peroxidase (SP) was used to perform the examination. Paraffin embedded sections with normal dehydration were performed antigen retrieval. The primary antibodies (Vimentin and E-cadherin), secondary antibody (mouse anti-rabbit IgG) and SP complex were dropwise added in sequence. Then we examined the expression levels of Vimentin and E-cadherin after 30 min incubation in 37 °C incubator. In 5 high power fields of each section, two experienced researchers independently analyzed and evaluated the levels of positive expression rates of Vimentin and E-cadherin, which were considered as yellow or brown granules appeared in the cells. The staining results were analyzed with staining intensity and positive cell number both taken into consideration. Immunoreactive score (IRS) was calculated by SI × PP. SI stands for staining intensity: 0 for no color, 1 for light yellow, 2 for brown, and 3 for dark brown. PP stands for the percentage of positive cells: 0 for no positive cells, 1 for less than 10%, 2 for 10 to 50%, 3 for 50 to 75%, and 4 for more than 75%. We set IRS ≤ 3 for negative and IRS > 3 for positive. Finally, the qualitative results of immunohistochemistry were determined by the overall conditions of the five random fields: Vimentin (+), Vimentin (−), E-cadherin (+), E-cadherin (−).

All of the experiment data were analyzed using SPSS 20 software. The analysis of enumeration data was performed using Fisher’s Exact test and the Spearman’s rank correlation was for the correlation analysis. *P* < 0.05 was considered significant.

## Results

**Fig. 1:**
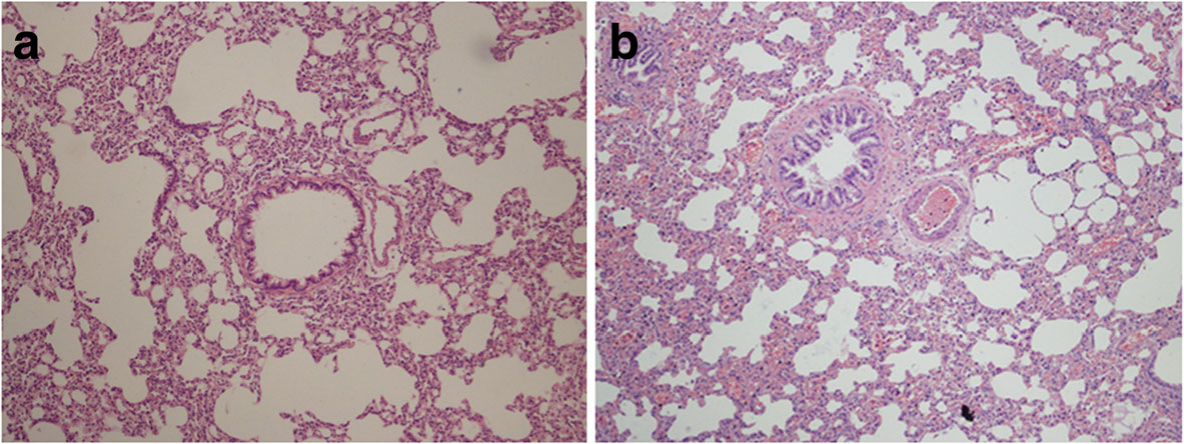
HE staining results of lung tissue sections in two groups. **a** Healthy lung in 100×; **b** transplanted lung in 100×

There were 13 rats for the successful modeling of experimental group and 8 sham-operated F344 rats for the control group. The HE staining results of tissue sections of healthy lungs and transplanted lungs were shown in Fig. 1. In the sections of healthy lungs, the wall of bronchiole had longitudinal plicae and were covered by pseudostratified ciliated columnar epithelium; there were no significant abnormality or just mild inflammatory cell infiltration that can be seen in histomorphology. On the contrary, the sections of transplanted lungs showed obviously abnormal bronchiole epithelium, which had hypertrophic mucosa and clear plicae. Peripheral inflammatory cell infiltration, stroma deposition and hyperplasia of fibrous tissue were found in submucosa. It was also visible for lamellar inflammatory exudate or even granulation tissues in the bronchiole lumen; and the lumen also had stenosis at different levels.

**Fig. 2:**
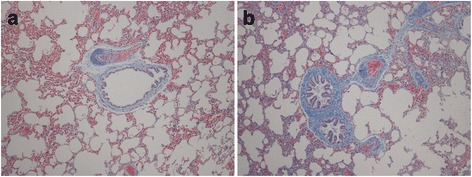
Masson staining results of lung tissue sections in two groups. **a** Healthy lung in 100×; **b** transplanted lung in 100×

The Masson staining sections of healthy and transplanted lung tissues were shown in Fig. 2. In the healthy lung tissue sections, the muscularis of bronchiole tunica media was thin and the smooth muscle was arranged in an undivided circle. However in the transplanted lung tissue sections, the muscularis of bronchiole tunica media became thick and the arrangement of smooth muscle was disordered. From there evident hyperplasia of fibrous tissue was also found.

**Fig. 3:**
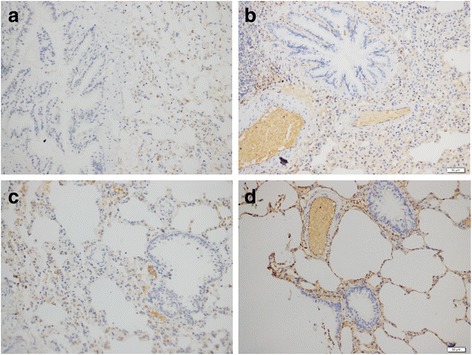
The expression of E-cadherin in lung tissue sections of the two groups; **a** transplanted lung, SI = 1, PP = 2, IRS = 2, E-cadherin (−); **b** transplanted lung, SI = 1, PP = 4, IRS = 4, E-cadherin (+); **c** healthy lung, SI = 1, PP = 3, IRS = 3, E-cadherin (−); **d** healthy lung, SI = 2, PP = 3, IRS = 6, E-cadherin (+). Notes: immunohistochemistry (200×); IRS for immunoreactive score, SI for staining intensity, PP for percentage of positive cells

**Fig. 4:**
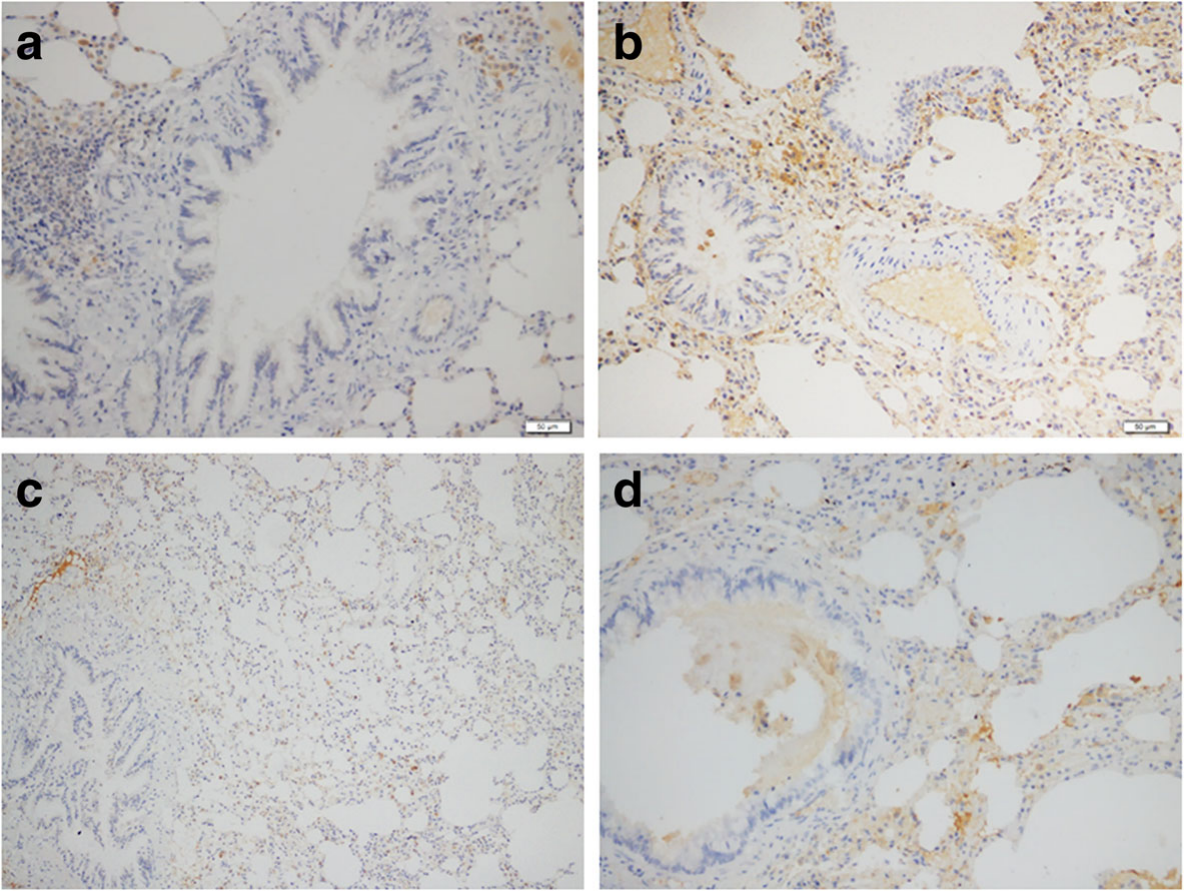
The expression of Vimentin in lung tissue sections of the two groups; **a** transplanted lung, SI = 2, PP = 1, IRS = 2, Vimentin (−); **b** transplanted lung, SI = 1.5, PP = 4, IRS = 6, Vimentin (+); **c** healthy lung, SI = 1, PP = 3, IRS = 3, Vimentin (−); **d** healthy lung, SI = 1, PP = 4, IRS = 4, Vimentin (+). Notes: immunohistochemistry (200×); IRS for immunoreactive score, SI for staining intensity, PP for percentage of positive cells

The immunohistochemistry results of E-cadherin and Vimentin expression were shown in Figs. 3 and 4. The overall qualitative result of 21 rats’ lung tissue specimens illustrated that there were 38.50% (5/13) of lung transplanted rats showed a positive expression of E-cadherin while it was 87.50% (7/8) of that in the healthy ones; and the difference between two groups was significant (*P* < 0.05). As for Vimentin, the positive expression rate of transplanted lung tissue sections was 76.92% (10/13) while in the healthy lung tissues it was 25.00% (2/8) and the significant difference existed between the two groups as well (*P* < 0.05). Spearman’s rank correlation analysis of the qualitative results of E-cadherin and Vimentin expression levels in the 21 lung tissue specimens demonstrated a negative correlation between them (*r* = −0.750, *P* < 0.01).

## Discussion

Lung transplantation (LT) is the only effective method in the treatment of terminal pulmonary disease, but the chronic dysfunction of transplanted lung remains to be a problem waiting to be solved with great urgency. According to its clinical manifestation and pathological characteristics, current studies believe that the chronic dysfunction is closely related to the self-inflammatory process, including bronchiolitis obliterans, chronic vascular rejection, post-transplantation large airway inflammation, chronic bronchiolitis, chronic lung stroma fibrosis and chronic pleurisy [14]. Among all of these, our study mainly discussed about the post-transplantation bronchiolitis obliterans.

Bronchiolitis obliterans is a chronic airway disease which mainly manifests as infiltration of airway neutrophils and macrophages. Conditions like oxidative stress and the imbalance of protease and antiprotease can lead to the release of many inflammatory mediators, such as TNF-α、IL-6、IL-8、IL-1. The inflammatory mediators finally causes airway mucus hypersecretion, airway smooth muscle hypertrophy, increased collagen synthesis and hyperplasia of airway microvessels, namely the airway remodeling. Airway remodeling is the key pathological change in the process of lung function decline caused by BO. In the transplanted lungs we found evidently abnormal bronchiole epithelium, which had hypertrophic mucosa and clear plicae. Peripheral inflammatory cell infiltration, stroma deposition and hyperplasia of fibrous tissue were found in submucosa. It was also visible for lamellar inflammatory exudate or even granulation tissue in the bronchiole lumen; and the lumen also had stenosis at different levels. These are the characteristics of airway remodeling and in accordance with pathological changes of BO. Therefore, we successfully established the model of post-lung transplantation BO.

In addition, we also explored the correlation between EMT and the airway remodeling. At present, many studies have demonstrated that EMT is associated with airway epithelial remodeling [15–17]. By observing a mouse asthma model, Johnson et al. [16] have found absence of E-cadherin expression, appearance of mesenchymal related indicators and thickening of smooth muscle in mouse airway epithelial cells after a 15-week exposure of dust mites. This work illustrated that EMT was involved in the process of asthma airway remodeling. The study of Sohal et al. [17] also demonstrated a close correlation between EMT and chronic obstructive pulmonary disease (COPD) airway remodeling. They found that cells in the cracks of basement membrane of COPD group expressed the mesenchymal cell markers such as S100A4, Vimentin and MMP9. Further immunohistochemistry analysis showed that there were correspondingly 13.8% and 7% of COPD sufferers’ basement epithelial cells and cells in reticular basement membrane simultaneously expressing keratin and S100A4, which means that EMT existed in the airway of COPD sufferers. Our immunohistochemistry results showed decreased E-cadherin expression and significantly increased Vimentin expression in transplanted lungs compared to normal lung tissues, which means an EMT process happened. Thus, the airway remodeling after lung transplantation is also related to EMT.

In our study, we noted that the BO pathological changes happened along with EMT in transplanted lung tissues, which testified EMT was involved in the small airway remodeling of BO. Inevitably, this study has a few limitations. The most important point among these is the relatively small sample size. In consideration of the complexity of the development and progress of BO after lung transplantation, small sample of a rat model has its natural drawbacks to present full understanding of such a huge issue. Secondly, although we tried to ensure that the experimental animal characteristics and the experimental conditions are similar, some common factors in animal model, such as various pathophysiological mechanisms, different responses to drugs, variations induced by techniques, differences in genetic regulation and anatomical differences, might have potential effects on this disease model. In addition, adding a Lewis to Lewis transplantation group as another control group would increase the reliability of the conclusions. Besides, it is also a pity that we still can’t identify the exact position of EMT in BO though they are existing simultaneously. We still don’t know whether that EMT is one of the causes of BO or it is only an accompanying phenomenon in the process of BO genesis and development. If EMT promotes the genesis and development of BO, then suppressing EMT may inhibit the progression of BO; while if EMT is just an accompanying phenomenon in the process of BO genesis and development, then further exploration of the mechanism of EMT is needed for the potential clues of post-lung transplantation BO.

## Conclusions

Although there were still some deficiencies, we believe that the established disease model in our study provided convincing evidences to prove that EMT is involved in the BO airway remodeling. However the role of EMT in BO still remains unknown and the specific relations between them is still remained to be explored. Further study of the mechanism and pathological significance of EMT in the transplanted lungs after lung transplantation may reveal novel targets for the prevention and treatment of post-lung transplantation bronchiolitis obliterans.

## Abbreviations

BO: Bronchiolitis obliterans
CLAD: Chronic lung allograft dysfunction
COPD: Chronic obstructive pulmonary disease
EMT: Epithelial-mesenchymal transition
HE: Hematoxylin-eosin
IgG: Immunoglobulin G
IL: Interleukine
IRS: Immunoreactive score
LT: Lung transplantation
MMP: Matrix metalloproteinase
MUC5AC: Mucin 5 AC
PP: Percentage of positive cells
SI: Staining intensity
SMA: Smooth muscle actin
SP: Streptavidin-peroxidase
SPF: Specific pathogen free
TNF: Tumor necrosis factor
